# A first report on prokaryotic diversity in northwestern Arafura deep-sea sediments, Indonesia

**DOI:** 10.1038/s41598-024-51614-6

**Published:** 2024-01-09

**Authors:** Yosmina Tapilatu, Ihsan Fauzan, Ariel Pradipta, Ali Budhi Kusuma

**Affiliations:** 1 Marine Microbiology and Biotechnology Laboratory, Centre for Deep-Sea Research, The National Research and Innovation Agency (PRLD BRIN), KKB Atjep Suwartana, Jl. Y. Syaranamual Guru-Guru Poka, Ambon, 97233 Indonesia; 2 Scientific Department, Genomik Solidaritas Indonesia (GSI Lab) Inc., Jl. Sultan Agung No. 29, South Jakarta, Indonesia; 3 Indonesian Centre for Extremophile Bioresources and Biotechnology, Faculty of Life Sciences and Technology, Sumbawa University of Technology (UTS), Jln. Raya Olat Maras, Desa Batu Alang, Moyo Hulu Sumbawa, 84371 Indonesia

**Keywords:** Biooceanography, Microbial ecology, Marine biology

## Abstract

Indonesia's deep-sea microbial communities remain poorly understood, prompting the need for comprehensive investigations. This study aimed to assess the bacterial and archaeal diversities in northwestern Arafura deep-sea sediments, spanning depths of 100 to 1,457 m using a 16S rRNA based-metagenomic sequencing approach, without technical and biological replicates. Principal component analyses based on the Bray–Curtis dissimilarity index indicated that most of the bacterial and archaeal communities were habitat-specific and influenced by depth. The most prevalent known bacterial phylotypes were detected from all samples belonging to the phylum of *Desulfobacteriota*, *Pseudomonadota*, and *Firmicutes*. In addition, the samples also harbored diverse members of the Archaea domain, including *Crenarchaeota*, *Nanoarchaeota* and *Haloarchaeota*. Notably, the sequencing data revealed the significant presence of rare prokaryotic taxa, including uncultured counterparts with less than 1% abundance. The findings suggest that novel and rare prokaryotic taxa are abundant in northwestern Arafura deep-sea ecosystem, offering unique opportunities for further bioprospecting and functional ecology studies.

## Introduction

Studying deep-sea microbial communities is crucial for understanding their role in marine ecology. However, information regarding Indonesian deep-sea microbial communities remains limited. Marine bacterial studies in Indonesia were more numerous in terms of quantity in the western area of the country^1^. In contrast, the eastern region, particularly its deep-sea environment, has received scant attention in scientific publications^2^. Previous studies in Indonesia primarily focused on the biotechnological potential of isolated bacteria, with minimal exploration of deep-sea microbial communities.

A significant milestone in deep-sea bacterial isolation occurred more than sixty years ago with a pioneering study analyzing sediment samples from the Banda Sea basin (7,250 m) as one of the stations^3^. Subsequent research conducted in the Banda Sea and adjacent areas has been scarce, if not absent, over the past seven decades^4^. The limited studies that were conducted focused predominantly on isolating potentially useful bacteria from coastal areas.

This study aims to address the existing knowledge gap regarding microbial communities and their potential ecological roles in the deep-sea environments of eastern Indonesia, with a particular emphasis on northwestern Arafura Sea. The Arafura Sea is a semienclosed ecosystem located between the Pacific and Indian Oceans and is surrounded by Indonesia, Australia, Timor Leste, and Papua New Guinea. The sea consists of waters covering the Arafura continental shelf between eastern Indonesia and northern Australia^5^. The Arafura Sea is bordered to the North by the Southern Seram Sea and to the South by the North Coast of Australia from the York Peninsula to the Don Peninsula. To the east, the Arafura Sea is limited by the Banda Sea and to the west by a line drawn from the Don Peninsula to Tanjung Aro Usu, the southwestern point of the Selaru Archipelago and Tanimbar^6^, whereas to the northwest, the Aru Trough divides the Arafura Sea from the Western Banda Sea. The sea is approximately 1,290 km in length and 560 km wide. Its depth on average is 80 m, and the maximum reaches approximately 3,400 m. The Arafura Sea hosts some of the world's highest marine biodiversity and contains some of the most pristine and highly threatened coastal and marine habitats^5^.

The Arafura Sea ecosystem is characterized by diverse habitats, such as coral reefs, mangroves, seagrass beds, sandy and muddy bottoms, and deep-sea sediments. These habitats support a rich variety of marine life, including fish, crustaceans, mollusks, echinoderms, marine turtles, cetaceans, dugongs, sharks, and rays. The sea is also an important fishing ground for the region, providing food security and livelihoods for millions of people^5^. However, the Arafura Sea ecosystem is facing serious threats from overexploitation, habitat degradation, pollution, and climate change. These pressures have resulted in declining fish stocks, coral bleaching, mangrove deforestation, invasive species, and reduced ecosystem resilience^5,7^.

Despite its ecological importance and vulnerability, the northwestern Arafura Sea ecosystem remains poorly studied and understood compared to its southern part. In particular, the microbial community composition of deep-sea sediment is largely unknown. Microbes play a crucial role in biogeochemical cycles and organic matter degradation in marine sediments and may also harbor novel metabolic pathways and bioactive compounds of potential biotechnological value. By exploring the diversity and function of the deep-sea microbes in the northwestern Arafura Sea, this research presents valuable insight to update our understanding of microbial communities in this region, which has been relatively understudied compared to other areas. It could shed new perspectives on the functioning and conservation of this unique ecosystem.

## Results

**Figure 1:**
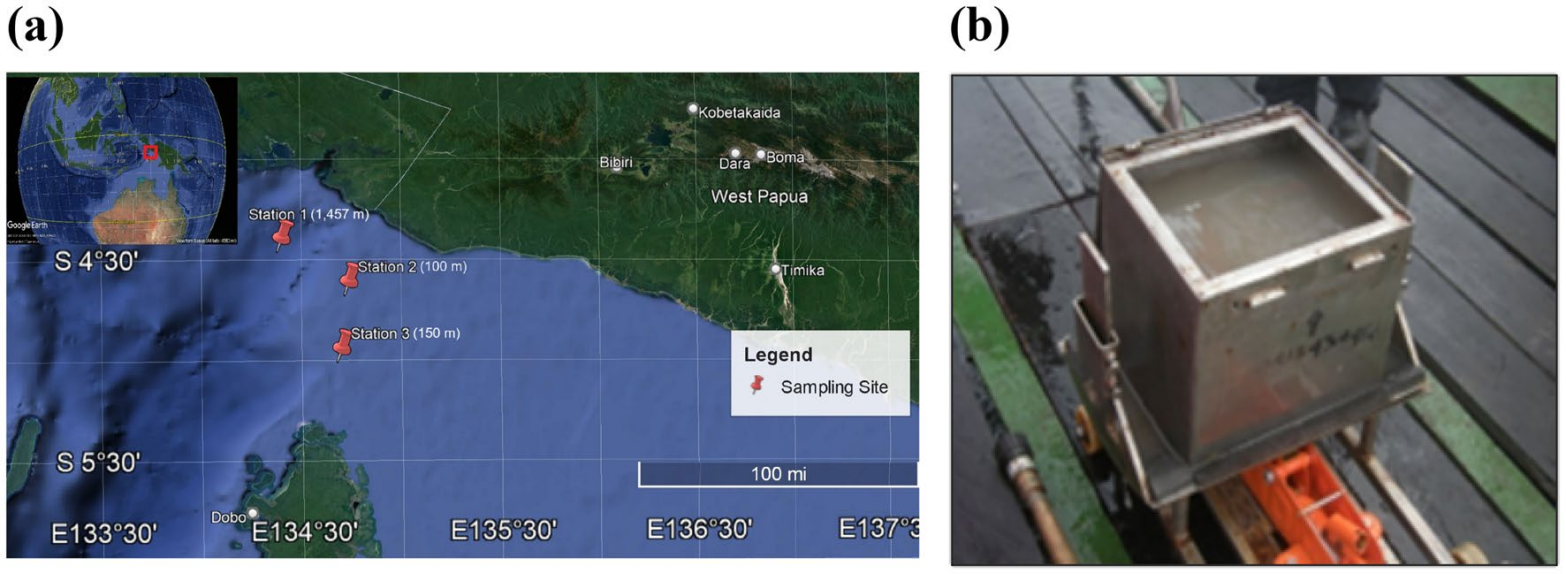
Sampling location and tool: (**a**) Sampling points in northwestern Arafura Sea were visualized using Google Earth Pro Version 7.3.6. Each station is indicated by a red pinpoint. (**b**) The sample was collected with an Ekman grab (pictured) connected to a steel sling controlled by a winch. Picture credit: Ihsan Fauzan (**a**); Yosmina Tapilatu (**b**).

We collected sediment samples from three different locations in the northwestern Arafura Sea at the boundary with the western Banda Sea in late February 2012 during the northwest monsoon period (Fig. 1a,b). Each location had a different depth: 100 m, 115 m, and 1,457 m, representing different environments (Suppl. Figure 2). The distance from Station 1 (1,457 m) to Station 2 (100 m) was about 64.4 km, and from Station 2 (100 m) to Station 3 (115 m) was about 55.6 km.

**Figure 2:**
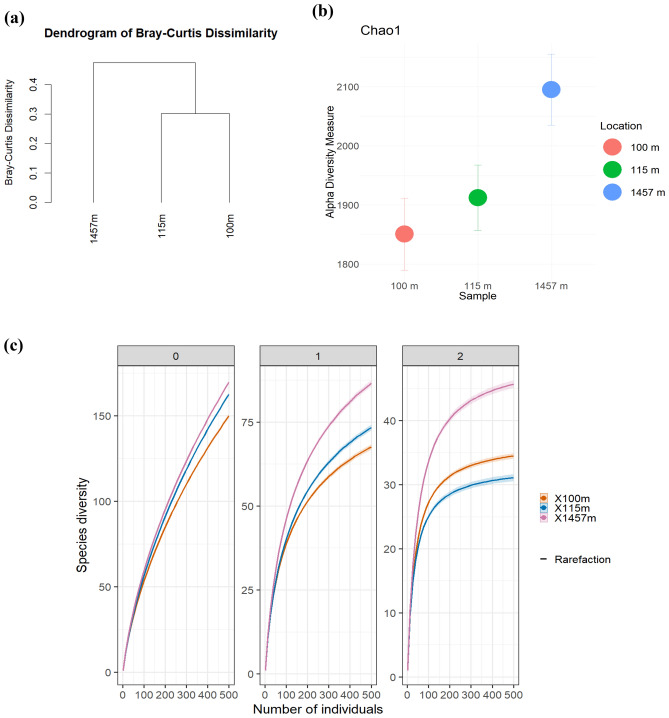
Bacterial and archaeal profile analysis of the Arafura deep-sea sediments for all depths: (**a**) Dendogram of Bray–Curtis Dissimilarity Index to show the distance between three different depths. (**b**) Chao1 index, error bars denote the standard error values, and (**c**) Hill’s numbers (Q0, Q1, Q2) analysis using observed sample of abundance to compute diversity estimates.

The Bray–Curtis Dissimilarity analysis revealed significant variations in the prokaryotic profiles across the three depths (1,457 m, 115 m, and 100 m), particularly when comparing the shallow depths (100 m and 115 m) to the 1,457 m depth (Fig. 2a). The Chao1 index analysis suggested a higher species richness at 1,457 m depth compared to the shallower depths (Fig. 2b). This difference was further supported by Hill’s number analysis (Q0, Q1 and Q2) (Fig. 2c). In terms of species richness (Q0), the sample from 1,457 m depth exhibited a richer set of OTUs than those from 115 and 100 m. The alpha diversity analysis (Q1, Shannon index) revealed higher diversity in the deeper area (1,457 m) compared to the shallower ones (100 m and 115 m) for both Bacteria and Archaea (Fig. 2c). Moreover, the samples from shallower depths displayed similar Simpson index values in both Bacteria and Archaea categories (Suppl. Table 1).

**Figure 3:**
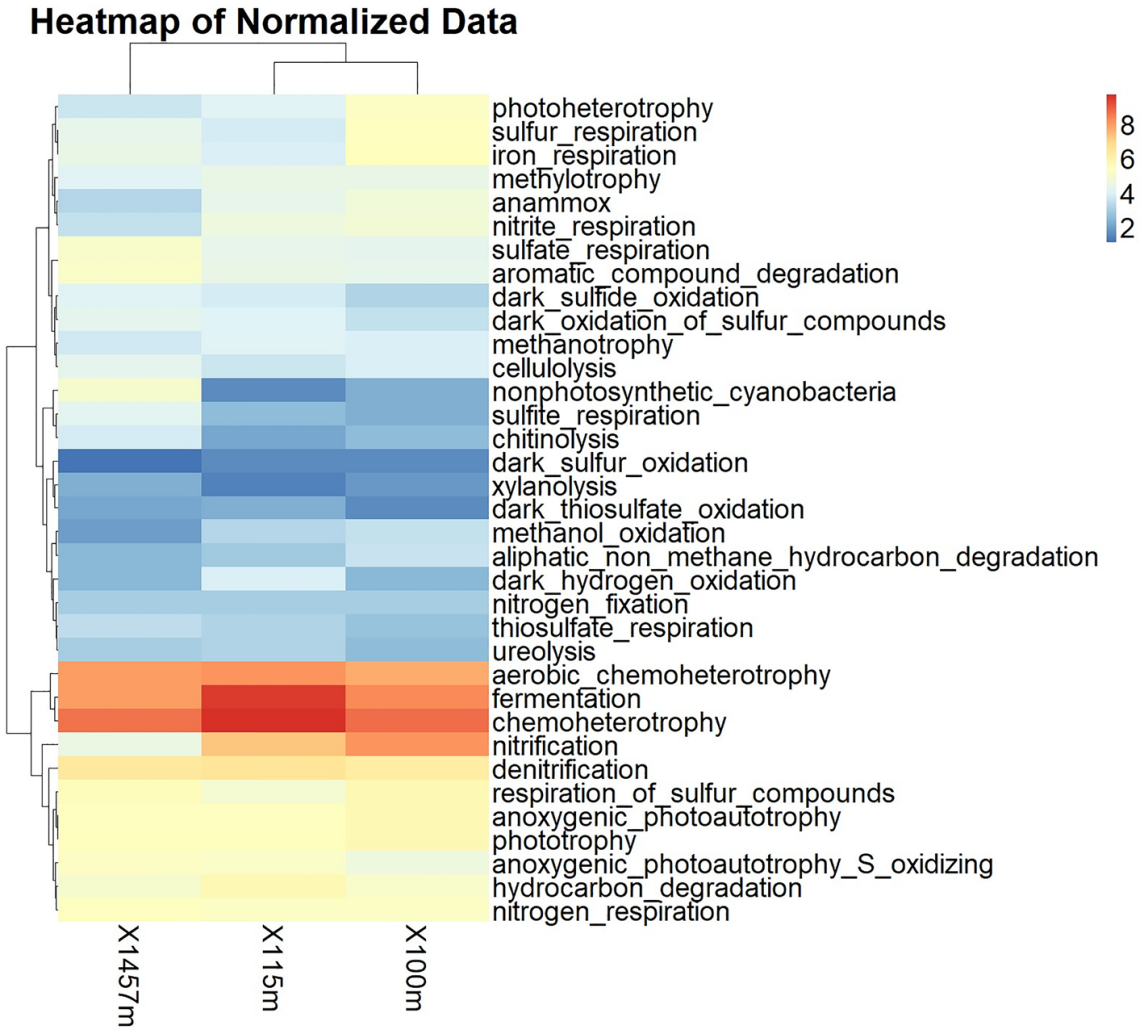
Heatmap plot regarding functional analysis based on taxonomic assignment using FAPROTAX. Values ​​are displayed on a scale on the graph. A lower value indicates fewer Operational Taxonomic Units (OTUs) associated with a specific function, while a higher value indicates more OTUs associated with that function.

The results of FAPROTAX analysis show various potential metabolic pathways in various samples at different depths (Fig. 3).

**Figure 4:**
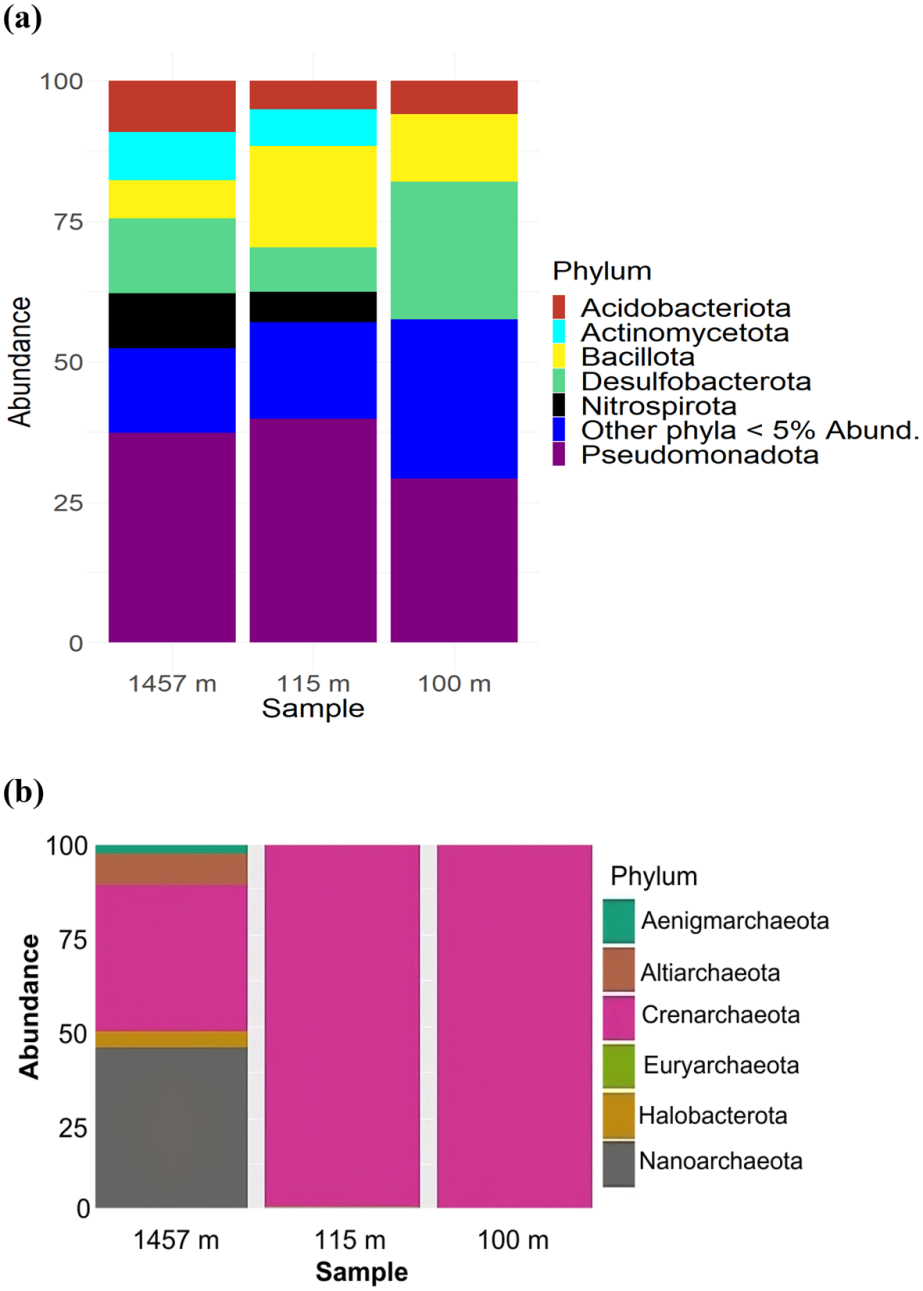
(**a**) Relative abundance analysis per phylum for Bacteria (**b**) Relative abundance analysis per phylum for Archaea. The abundance value is presented as a percentage (%).

Microbial profiles at each depth were analyzed for bacterial relative abundance (Fig. 4a). *Pseudomonadota* (previously *Proteobacteria*) was the predominant bacterial phylum at each depth, with a relative proportion of more than 30%. *Desulfobacteria* was the second most abundant (~ 28%) at 1,457 m. Additionally, *Bacillota* (previously *Firmicutes*) composition varied at each depth, with a higher proportion (~ 26%) at 115 m than at 100 m (~ 9%) and 1,457 m (~ 10%). In addition to visible bacterial profile differences, noticeable phylum shifts occurred in the Archaea community at 1,457 m (Fig. 4b). While *Crenarchaeota* was highly dominant at shallow depths, *Nanoarchaeota* comprised over 32% of the community at 1,457 m.

At the genus level, JTB255 Marine Benthic Group (JTB255-MBG) and *Pseudomonas* were the two predominant genera (more than 3%) from the *Pseudomonadota* group at 1,457 m. Further composition analysis indicated that *Lactobacillus* was the predominant (> 75%) genus at 115 m relative to all phyla in *Bacillota* (Suppl. Figure 1b).

## Discussion

The environmental parameters of the northwestern Arafura Sea were measured in 2012 and 2014^8^, revealing similar profiles in temperature and salinity at 100 and 115 m depth with the surface and different profiles at 600 m (Suppl. Figure 2). In terms of nutrients, increased nitrate concentration at 1,000 m^8^ was likely caused by Papua's southwest coast river discharge^9^, as well as from the bordering Banda Sea during the NW monsoon period^10^. In contrast, oxygen concentration was decreased to 3 mg l^−1^ at the same depth, which was likely caused by microbial decomposition^11,12^.

Exposure to different water masses at varying depths influences the nutrient and organic matter contents, thereby affecting the distribution and composition of marine bacteria. Previous studies have identified vertical water mass segregation as a key factor in determining bacterial community structure^13–15^.The above environmental profile surely plays a role in bacterial and archaeal community compositions among different depths, with higher microbial diversity observed at greater depths. These findings are consistent with previous studies conducted in the Red Sea^16^, where prokaryotes communities were more diverse at deeper points (> 200 m) than at shallower depths (0–200 m). The dominant phyla identified in this study (*Pseudomonadota*) are similar to the results reported from the North Atlantic^17^ and Bay of Bengal^18^, indicating their wide distribution and potential functional roles. The higher abundance of *Desulfobacteria* at 1,457 m suggests its significant involvement in biogeochemical cycles, particularly sulfate reduction in anaerobic conditions^19^. However, this comparison does not consider the same taxonomic level as that carried out in previous studies due to the different methods used. In addition, these previous studies have different environmental conditions that might influence diversity.

The lack of replicate samples due to several constraints (cf. Methods section, sample collection and storage) is a caveat in our study and restrains the interpretation of our data. Nonetheless, the diversity indices (Fig. 2) indicated the quite distinct feature of the three stations prokaryotic communities. The difference in prokaryotic diversity among the three sampling locations can also be partially attributed to the presence of two phyla (*Aenigmarchaeota* and *Nanoarchaeota*) belonging to the DPANN archaeal superphylum at 1,475 m. *Nanoarchaeota* dominated the archaeal assemblage in this sediment sample. The DPANN superphylum contains archaea with extremely small cell and genome sizes and limited metabolic capabilities^20^. These identified taxa at 1,475 m may suggest dependent symbiotic interactions with other organisms, as has been previously described^21^. The shifts in Archaea phyla at 1,457 m, with the occurrence of *Nanoarchaeota*, were observed previously in the North Atlantic^22^.The presence of this archaea phyla was also featured from the three deep-sea hydrothermal vent sites: the Eastern Lau Spreading Center (M10-121), Guaymas Basin (Gua-46) and the Mid-Cayman Rise (MC-1) following the shot-gun metagenomic surveys^23^. This highlights the need for a deeper understanding of their functional contributions in the deep sea. *Crenarchaeota*, was reported to play a crucial role in the marine nitrogen cycle^24^. While *Nanoarchaeota* has been found in deep-sea hydrothermal vents, its functional contribution in the deep sea remains poorly analyzed^23^.

The *Pseudomonadota* phyla (Suppl. Figure 1c) showed the presence of four genera known to be involved in the nitrogen cycle at different depths. Two genera previously reported as part of the ammonia-oxidizing bacteria group^25^ (AqS1 and Cm1-21) were detected at 110 and 115 m but were absent at 1,457 m. Conversely, two other genera (JTB255-MBG and *Pseudomonas*) involved in the denitrification pathway^26^, were present at 1,457 m. This suggests their potential role in efficient nitrogen removal and transformation, influencing overall nitrogen cycling dynamics. This aligns with the FAPROTAX analysis, confirming *Pseudomonadota*’s role in organic matter decomposition in the deep-sea ecosystem^27^. Additionally, the presence of *Coxiella* was noted at 115 and 1,457 m. This genus was previously reported to be present in at least four data sets of marine amplicons, from seawater and deep-sea sediments, including samples from adjacent Australian marine benthic and pelagic zones, the Barents Sea (Norway), Tara Ocean DNA samples and eDNAbyss sediment samples^28^.

Several genera (*Dialister*, *Gemella*, *Veilonella,* and *Lactobacillus*) from the *Firmicutes* phylum (Suppl Fig. 1b) were also present in the previously mentioned four data sets^28^. These genera, typically associated with land mammals, could be present due to potential contamination during sample handling or kit contaminants. Alternatively, their presence may be linked to terrestrial organic matter input from the Papua mainland, as evidenced by seabed sediment core studies^29^. This suggests that the Arafura Sea could be a sink for terrestrial nutrients from Papua Island^30^. The northwestern area of the Arafura Sea, where the sediment samples were collected (Fig. 1b), is an upwelling hotspot^9^ in the Maluku Province marine environment. Nutrient enrichment from West Papua River runoffs, particularly during the southeast monsoon, contributes to high primary productivity in the sea surface layer^31^. This area, heavily impacted by both international and local fishing activities supports a diverse range of organisms and microorganisms due to its nutrient-rich environment, thereby positively impacting biodiversity^32^. Nevertheless, further studies are needed to understand nutritional sources, as anthropogenic factors can cause ecological imbalances^33^, particularly in deep-sea ecosystems.

Given the remoteness of deep-sea ecosystems, our capacity to successfully collect and analyze the inhabiting microorganisms will be critical, particularly in the case of the study's location in Indonesia. A lack of proper research infrastructure and funding hampered this type of endeavor. This constraint, in addition to revealing the preliminary essence of our findings, revealed the necessity for additional research into less-explored deep-sea locations such as the Arafura Sea. Because the study's results are currently based on the examination of only three separate samples without the presence of replicates, either technical or biological, additional samples are necessary to corroborate the findings of this assessment.

Overall, this study reveals varying microbial proportions across depths, particularly at shallower depths (100 m and 115 m) versus deeper areas (1,457 m), showcasing higher diversity in the latter. This finding is substantiated by the previous report on archaea diversity studies across the depth above 2000 m in The South China Sea^34^. These depth-dependent differences in microbial taxa may impact their involvement in biogeochemical cycles (e.g., nitrogen, sulfur, and carbon)^35^. This highlights the significant role of deep ocean (> 1000 m) microbes in biogeochemical cycles, prompting further exploration of their advanced functional properties and key genes using techniques such as shotgun metagenomics^36^.

## Methods

### Study area description

The sampling locations in the northern Arafura Sea were located between Aru Island in the south and West Papua Province in the north. Overall, the Arafura Sea was chosen as the sampling location to study the changes occurring in its environment, including the cause of fisheries stock decrease in the area, as a follow-up from local authorities’ request. Station 1 was situated in the Aru Trough, which explained the depth reached (1,457 m). The other two stations were in the proximities of West Papua Province.

### Sample collection and storage

Bottom surface sediments were collected onboard Research Vessel Baruna Jaya VII (R/V BJ VII) during a ten-day cruise conducted between February 25th and March 05th, 2012, as a parallel expedition of an externally funded Environmental Baseline Assessment. A grab sampler (Fig. 1c) was deployed at depths of 100, 115, and 1,457 m. Several constraints, i.e., time, R/V infrastructures, and underwater currents, led to the deployment of only one grab sampler per station; thus, no biological replicate was available. Subsamples of 500 g to 1 kg were stored at − 20 °C onboard and in the lab. Environmental parameters (conductivity, temperature, salinity, turbulence, and sigma-t) were measured in situ using a CTD SBE 911 + (Sea-Bird Scientific) mounted on a rosette with Niskin bottles. Due to time constraints and strong currents, these parameter measurements were performed only at the surface, middle, and lowest points reachable with the functioning crane.

### Sample preparation and sequencing

Samples (~ 1 g) of sediment from each location underwent environmental DNA (eDNA) extraction using the PowerSoil DNA extraction kit (Qiagen, Germany). Subsequent preparation and sequencing were performed by the sequencing service company FirstBase (Singapore). The sequencing process did not include a control sample. The V4 region of the 16S rRNA gene was amplified using F515/R806 primers (5'-GTGCCAGCMGCCGCGGTAA-3'; 5'-GGACTACHVGGGTWTCTAAT-3') with barcodes, and Phusion® High-Fidelity PCR Master Mix (New England Biolabs) was used for all PCRs.

Sequencing libraries were generated using the TruSeq® DNA PCR-Free Sample Preparation Kit (Illumina, USA) following the manufacturer's recommendations, and index codes were added. The library quality was assessed on the Qubit@ 2.0 Fluorometer (Thermo Scientific) and Agilent Bioanalyzer 2100 system. Amplicon metagenomic sequencing was conducted on an Illumina HiSeq 2500 platform, generating 250 bp paired-end reads.

### Data analysis

The output data analysis and annotation were performed using the Kraken2 data analysis package^37^. We chose to use Kraken2 because it is up to 300 times faster and more accurate at 16S profiling than other tools^38^. Raw sequences were filtered according to base quality score, average base content per read and GC distribution in the reads using Cutadapt v.4.4^39^. Filtered sequences were taxonomically assigned using Kraken2 with SILVA v.138.1 database and generated a kraken2 report as output. Then, OTUs and read counts were extracted from the report using kraken-biom and saved as a biom format file^40^. Biom files generated from this process were then subjected to abundance and diversity analysis using the phyloseq version 1.44.0 package from RStudio version 2023.3.0.386^41^. Abundance information was normalized using a standard sequence number corresponding to the sample with the fewest sequences. Subsequent analyses of Bray–Curtis Dissimilarity, Chao1 Index, and Hill’s Number (Q0-2) were all performed based on these output normalized data. Functional analysis was performed using FAPROTAX version 1.7.3^42^. Biom files generated from the Kraken2 process were used as input for FAPROTAX analysis. The result obtained was in.txt format, information regarding total records for each functional group assignment was then extracted, and normalized using natural logarithmic, and a heatmap was visualized using pheatmap package from RStudio version 2023.3.0.386. All bash and R scripts used in this study are provided in the supplementary documentation.

### Supplementary Information

Supplementary Information.

## Data Availability

Raw amplicon reads are deposited at the NCBI under BioProject PRJNA979004, accessible at https://www.ncbi.nlm.nih.gov/bioproject/PRJNA979004, and at the Indonesian Scientific Repository (RIN), accessible at https://data.brin.go.id/dataset.xhtml?persistentId=hdl:20.500.12690/RIN/TNFKG4.
