# Supplementary material for: A first report on prokaryotic diversity in northwestern Arafura deep-sea sediments, Indonesia
Source: Sci Rep. 2024 Jan 9;14:895. doi: 10.1038/s41598-024-51614-6 (PMC10776683; doi:10.1038/s41598-024-51614-6)
Supplement: Supplementary file 1 — Supplementary Information. [file 41598_2024_51614_MOESM1_ESM.pdf]

A first report on prokaryotic diversity in northwestern Arafura deep-sea sediments, Indonesia

Yosmina Tapilatu[1,*], Ihsan Fauzan[2], Ariel Pradipta[2], and Ali Budhi Kusuma[3]

[1]Marine Microbiology and Biotechnology Laboratory, Centre for Deep-Sea Research, The National Research and Innovation Agency (PRLD BRIN), KKB Atjep Suwartana, Jl. Y. Syaranamual Guru-guru Poka Ambon 97233, Indonesia

[2]Scientific Department, Genomik Solidaritas Indonesia (GSI Lab) Inc., Jl. Sultan Agung No. 29, South Jakarta, Indonesia

[3]Indonesian Centre for Extremophile Bioresources and Biotechnology, Faculty of Life Sciences and Technology, Sumbawa University of Technology (UTS), Jln. Raya Olat Maras, Desa Batu Alang, Moyo Hulu Sumbawa, 84371, Indonesia

*Corresponding author: yosmina.tapilatu@brin.go.id; yosmina.lipi.ambon@gmail.com

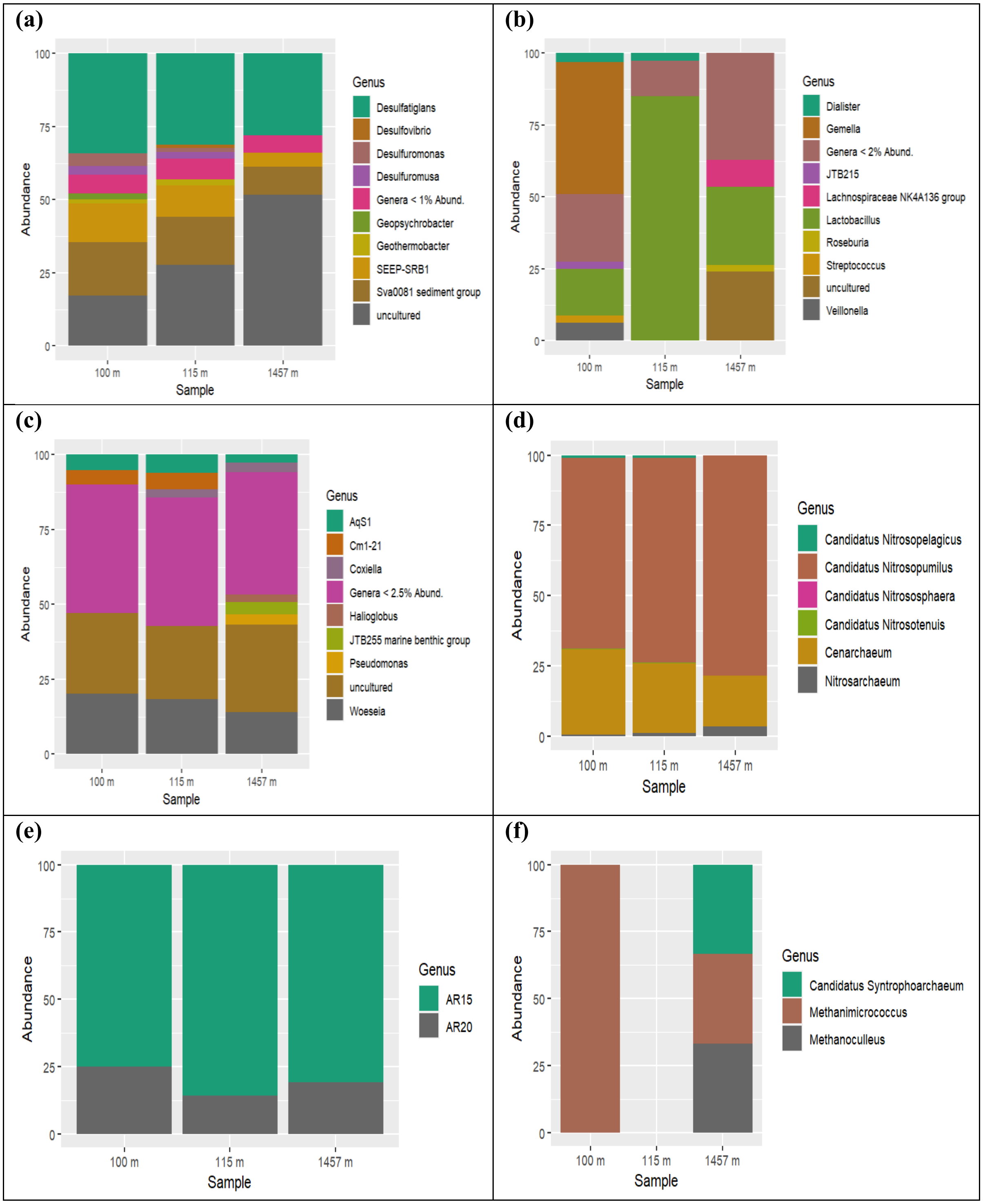

Supplementary Figure 1. Prokaryote Profile Analysis of Arafura Deep Sea per Genus Level (a) *Desulfobacteriota* phyla profile per genus level (b) *Firmicutes* phyla profile per genus level (c) *Pseudomonadota* phyla profile per genus level (d) *Crenarchaeota* phyla profile per genus level (e) *Nanoarchaeota* phyla profile per genus level (f) *Haloarchaeota* phyla profile per genus level.

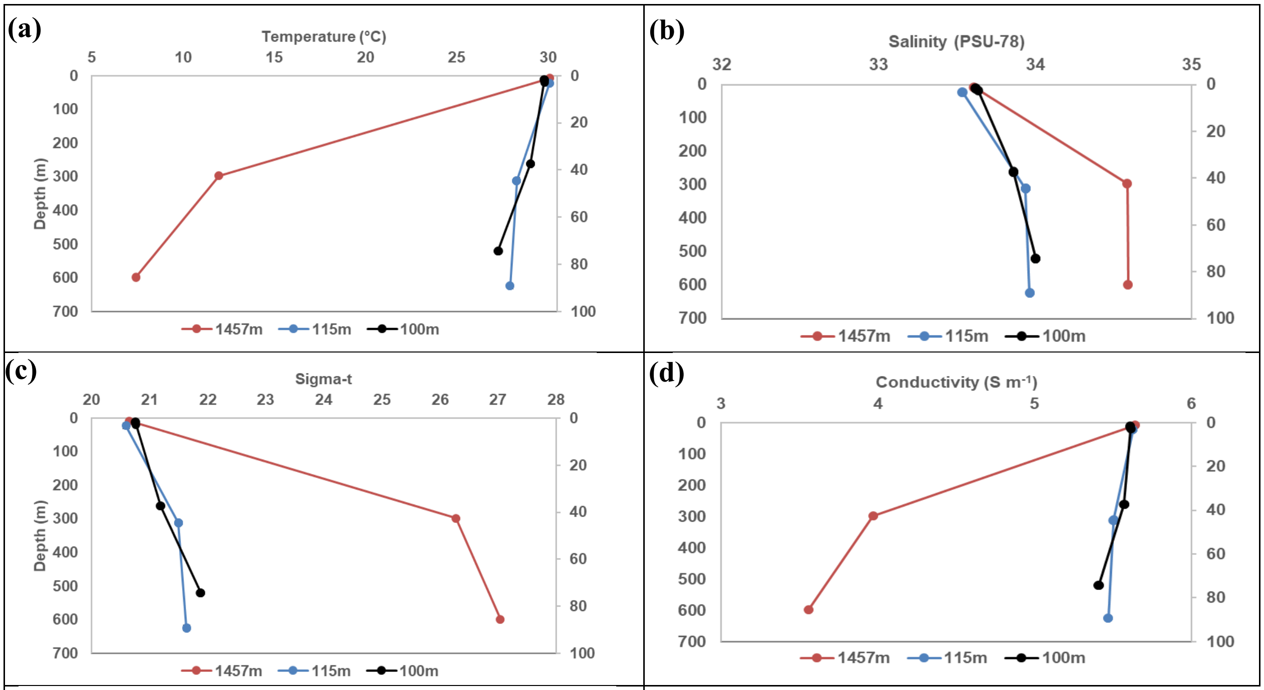

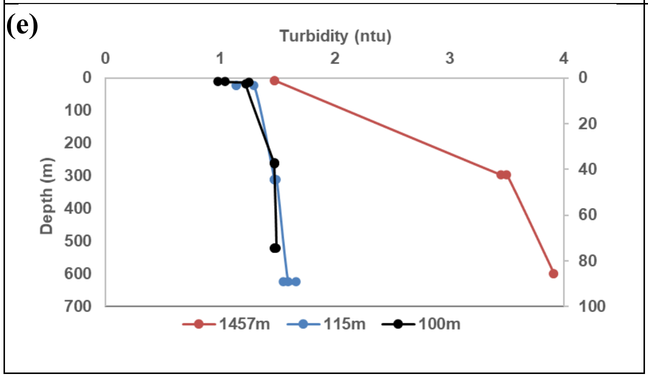

Supplementary Figure 2. The measurement result of (a) temperature (b) salinity (c) sigma-t (d) conductivity and (e) turbidity during sediment sampling, using CTD SBE 911+, done on all locations only on three layers: the surface, middle, and lowest point possible. The secondary y-axis also indicates depth (m) for 100m and 115m data sets versus those of 1,457m plotted at the primary one.

Supplementary Table 1. Detailed information on environment parameters, archaea, and bacteria diversity at each sampling stations

**Station**	**1**	**2**	**3**
	**Environmental condition**		
Position (Lat.,Long.)	-4.387, 134.409	-4.601, 134.743	-4.946, 134.705
Depth (m)	1 457	100	115
Texture	Muddy sand	Muddy sand	Muddy sand
	**Archaea**		
Total Abundance	304	7318	4563
Number of OTU	27	23	25
Simpson	0.87	0.52	0.39
Shannon	2.47	1.00	0.83
	**Bacteria**		
Total Abundance	88804	76313	83933
Number of OTU	1590	1367	1467
Simpson	0.98	0.97	0.97
Shannon	4.96	4.67	4.77

```
#Script 3 for all analyses in R

# Load required packages, input data from kraken-biom result
if (!requireNamespace("BiocManager", quietly = TRUE))
  install.packages("BiocManager")
BiocManager::install("phyloseq") # Install phyloseq
install.packages(c("ggplot2", "readr", "patchwork")) #install ggplot2 and patchwork
to chart publication-quality plots and readr to read rectangular datasets.
library("phyloseq") # load library
library("phyloseq") # load library
library("ggplot2") #load library
library("readr") #load library
library("patchwork") #load library

# Load data with the number of reads per OTU and taxonomic labels for each OTU
setwd("C:/Users/Ihsan Fauzan/Documents/Paper/") # tell R in which directory we are
working
all_biomdata <- import_biom("all.biom")
class(all_biomdata) # The "class" command indicate that we already have our
phyloseq object
View(all_biomdata@tax_table@.Data)
View(all_biomdata@otu_table@.Data)

#MENGUBAH NAMA SAMPEL
library(phyloseq)
library(biomformat)

# Mengubah nama sampel pada objek phyloseq
new_sample_names <- c("115 m", "1457 m", "100 m")
sample_names(all_biomdata) <- new_sample_names

# Adjustment penamaan
all_biomdata@tax_table <- substring(all_biomdata@tax_table, 4) # detele the 4
letter of tax_table
colnames(all_biomdata@tax_table)<- c("Kingdom", "Phylum", "Class", "Order",
                                     "Family", "Genus", "Species") # rename the
"Rank" with the taxonomic order

# Using glom ###
glom <- tax_glom(all_biomdata_bacteria, taxrank='Genus')
glom_phylum <- tax_glom(all_biomdata_bacteria, taxrank = "Phylum")

# Convert to csv
dat <- psmelt(glom)
dat2 <- psmelt(all_biomdata_bacteria)
dat_phylum <- psmelt(glom_phylum)

write.csv(dat, file='C:/Users/Ihsan Fauzan/Documents/Paper/bacteria.csv')
write.csv(dat_phylum, file = "C:/Users/Ihsan
Fauzan/Documents/Paper/archaea_phylum.csv")
```

```
# Counting phyla
unique(all_biomdata@tax_table@.Data[,"Phylum"])

#EXPLORING ABUNDANCE TABLE
View(all_biomdata@otu_table@.Data)
write.csv(all_biomdata@otu_table@.Data, file = "C:/Users/Ihsan
Fauzan/Documents/Paper/rawcount_all.csv")

#ALPHA DIVERSITY PLOT
all_biomdata #Checking OTU and Taxonomy Table
sample_sums(all_biomdata) #Count total sample for each group
summary(all_biomdata@otu_table@.Data)

richness_diversity <- estimate_richness(all_biomdata_bacteria, measures =
c("Observed", "Shannon","Simpson"))
View(richness_diversity)

richness_diversity_df <- as.data.frame(richness_diversity)
richness_diversity_df

write.csv(richness_diversity, file = "C:/Users/Ihsan
Fauzan/Documents/Paper/Alpha_Bacteria.csv")

plot_richness(physeq = all_biomdata,
              measures = c("Shannon","Simpson"))
#RAREFACTION CURVE ANALYSIS
library(vegan)

richness <- estimate_richness(all_biomdata, measures = "Observed")
estimate_richness(all_biomdata, measures = "Shannon")

rarecurve(all_biomdata, step = 100, sample = min(richness), col = c("red","blue"),
label = TRUE)

#Absolute and Relative Abundances
summary(all_biomdata@tax_table@.Data== "")

all_biomdata_1 <- subset_taxa(all_biomdata, Genus != "")
summary(all_biomdata_1@tax_table@.Data== "")
head(all_biomdata_1@otu_table@.Data)

percentages <- transform_sample_counts(all_biomdata_1, function(x) x*100 / sum(x) )
head(percentages@otu_table@.Data)

#BETA DIVERSITY
distanceMethodList
metadata <- data.frame(Sample=c("115 m", "1457 m", "100 m"), Location=c("115 m",
"1457 m", "100 m")) # Making dataframe with metadata
```

```
#NMDS Plot
View(metadata)
rownames(metadata) <- metadata$Sample # Using sample names as row names
percentages@sam_data <- sample_data(metadata) # Adding metadata to sam_data table
of phyloseq object percentages

meta_ord <- ordinate(physeq = percentages, method = "NMDS", distance = "bray") #
Calculating beta diversity
plot_ordination(physeq = percentages, ordination = meta_ord, color = "Location") #
Plotting beta diversity

#PCoA Plot
pcoa <- ordinate(physeq = percentages, method = "PCoA", distance = "bray")
plot_ordination(physeq = percentages, ordination = pcoa, color = "Location")

#EXPLORING DEEPER

percentages_glom <- tax_glom(percentages, taxrank = 'Genus')
View(percentages_glom@tax_table@.Data)
percentages_df <- psmelt(percentages_glom)
str(percentages_df)
View(percentages_df)
write.csv(percentages_df, file = "C:/Users/Ihsan
Fauzan/Documents/Paper/all_phylum_2.csv")

absolute_glom <- tax_glom(physeq = all_biomdata_1, taxrank = "Genus")
absolute_df <- psmelt(absolute_glom)
View(absolute_df)
str(absolute_df)

absolute_df$Genus <- as.factor(absolute_df$Genus)

#Install RColorBrewer dan Load bagi yang belum ada
install.packages("RColorBrewer")
library(RColorBrewer)

#Plotting lanjut
colors_abs <-
colorRampPalette(brewer.pal(8,"Dark2"))(length(levels(absolute_df$Genus)))

absolute_plot <- ggplot(data= absolute_df, aes(x=Sample, y=Abundance, fill=Genus))+

  geom_bar(aes(), stat="identity", position="stack")+
  scale_fill_manual(values = colors_abs)

percentages_df$Genus <- as.factor(percentages_df$Genus)
phylum_colors_rel <- colorRampPalette(brewer.pal(8,"Dark2"))
(length(levels(percentages_df$Genus)))
relative_plot <- ggplot(data=percentages_df, aes(x=Sample, y=Abundance,
fill=Kingdom))+
```

```
  geom_bar(aes(), stat="identity", position="stack")+
  scale_fill_manual(values = phylum_colors_rel)

absolute_plot #Loading the plot
relative_plot #Loading the plot

#UNTUK EFISIENSI CUTOFF X%
percentages_df$Genus <- as.character(percentages_df$Genus)
percentages_df$Genus[percentages_df$Abundance < 2.5] <- "Phyla < 2.5% abund."
unique(percentages_df$Genus)

percentages_df$Genus <- as.factor(percentages_df$Genus)
phylum_colors_rel<- colorRampPalette(brewer.pal(8,"Dark2"))
(length(levels(percentages_df$Genus)))
relative_plot <- ggplot(data=percentages_df, aes(x=Sample, y=Abundance,
fill=Genus))+
  geom_bar(aes(), stat="identity", position="stack")+
  scale_fill_manual(values = phylum_colors_rel)

relative_plot

#UNTUK LEBIH LENGKAP DENGAN VALUE MASING-MASING PADA STACK BAR
relative_plot <- ggplot(data=percentages_df, aes(x=Sample, y=Abundance,
fill=Phylum))+
  geom_bar(aes(), stat="identity", position="stack")+
  geom_text(aes(label = Abundance), position = position_stack(vjust = 0.5)) + #
menambahkan layer untuk menampilkan nilai Abundance pada setiap bar
  scale_fill_manual(values = phylum_colors_rel)

relative_plot

View(percentages_df)
write.csv(percentages_df, file = "C:/Users/Ihsan
Fauzan/Documents/Paper/bacteria_phylum.csv")
View(percentages@otu_table@.Data)
View(all_biomdata_bacteria_1@tax_table@.Data)

#PLOT PER TAXA
#Plot Crenarchaeota
Desul <- subset_taxa(all_biomdata_bacteria_1, Phylum == "Firmicutes")
unique(Desul@tax_table@.Data[,2])

Desul_percentage <- transform_sample_counts(Desul,function(x) x*100 / sum(x))

#KALO MAU DI GLOM
Desul_glom <- tax_glom(Desul_percentage, taxrank = "Genus")
Desul_df <- psmelt(Desul_glom)

#KALAU TIDAK MAU DI GLOM
Desul_df <- psmelt(Desul_percentage)
```

```
#UNTUK PLOTTING
Desul_df$Genus[Desul_df$Abundance < 2] <- "Genera < 2% Abund."
Desul_df$Genus <- as.factor(Desul_df$Genus)
genus_color_Desul <-
colorRampPalette(brewer.pal(8,"Dark2"))(length(levels(Desul_df$Genus)))

Plot_Desul <- ggplot(data = Desul_df, aes(x=Sample, y=Abundance, fill=Genus))+
  geom_bar(aes(), stat = "identity", position = "stack")+
  scale_fill_manual(values = genus_color_Desul)
Plot_Desul

#JUST FOR CHECKING
View(Desul_df)
View(Desul_percentage@tax_table@.Data)
View(Desul_glom@tax_table@.Data)
View(all_biomdata_bacteria_1@tax_table@.Data)
```

```
#Script for Bacteria Analysis in R

# Load required packages, input data from kraken-biom result
if (!requireNamespace("BiocManager", quietly = TRUE))
  install.packages("BiocManager")
BiocManager::install("phyloseq") # Install phyloseq
install.packages(c("ggplot2", "readr", "patchwork")) #install ggplot2 and patchwork
to chart publication-quality plots and readr to read rectangular datasets.
library("phyloseq") # load library
library("phyloseq") # load library
library("ggplot2") #load library
library("readr") #load library
library("patchwork") #load library

# Load data with the number of reads per OTU and taxonomic labels for each OTU
setwd("$PATH") # tell R in which directory we are working
all_biomdata <- import_biom("all.biom")
class(all_biomdata) # The "class" command indicate that we already have our
phyloseq object
View(all_biomdata@tax_table@.Data)
View(all_biomdata@otu_table@.Data)

#Load another package
library(phyloseq)
library(biomformat)

# Add sample name
new_sample_names <- c("115 m", "1457 m", "100 m")
sample_names(all_biomdata) <- new_sample_names

# Adjustment of taxa name
all_biomdata@tax_table <- substring(all_biomdata@tax_table, 4) # detele the 4
letter of tax_table
colnames(all_biomdata@tax_table)<- c("Kingdom", "Phylum", "Class", "Order",
                                     "Family", "Genus", "Species") # rename the
"Rank" with the taxonomic order

all_biomdata_bacteria <- subset_taxa(all_biomdata, Kingdom == "Bacteria") # Filter
all data samples for Bacteria
View(all_biomdata_bacteria@tax_table@.Data)
View(all_biomdata_bacteria@otu_table@.Data)

# Using glom
glom <- tax_glom(all_biomdata_bacteria, taxrank='Genus')
glom_phylum <- tax_glom(all_biomdata_bacteria, taxrank = "Phylum")

# Convert to csv
dat <- psmelt(glom)
dat2 <- psmelt(all_biomdata_bacteria)
dat_phylum <- psmelt(glom_phylum)
```

```
write.csv(dat, file='$PATH')
write.csv(dat_phylum, file = "$PATH")

# Counting phyla
unique(all_biomdata_bacteria@tax_table@.Data[,"Phylum"])

#EXPLORING ABUNDANCE TABLE
View(all_biomdata_bacteria@otu_table@.Data)
write.csv(all_biomdata_bacteria@otu_table@.Data, file = "$PATH")

#ALPHA DIVERSITY PLOT
all_biomdata_bacteria #Checking OTU and Taxonomy Table
sample_sums(all_biomdata_bacteria) #Count total sample for each group
summary(all_biomdata@otu_table@.Data)

richness_diversity <- estimate_richness(all_biomdata_bacteria, measures =
c("Observed", "Shannon","Simpson"))
View(richness_diversity)

richness_diversity_df <- as.data.frame(richness_diversity)
richness_diversity_df

write.csv(richness_diversity, file = "$PATH")

plot_richness(physeq = all_biomdata_bacteria,
              measures = c("Shannon","Simpson"))

#Absolute and Relative Abundances
summary(all_biomdata_bacteria@tax_table@.Data== "")

all_biomdata_bacteria_1 <- subset_taxa(all_biomdata_bacteria, Genus != "")
summary(all_biomdata_bacteria_1@tax_table@.Data== "")
head(all_biomdata_bacteria_1@otu_table@.Data)

percentages <- transform_sample_counts(all_biomdata_bacteria_1, function(x) x*100 /
sum(x) )
head(percentages@otu_table@.Data)

#BETA DIVERSITY
distanceMethodList
metadata <- data.frame(Sample=c("115 m", "1457 m", "100 m"), Location=c("115 m",
"1457 m", "100 m")) # Making dataframe with metadata

#NMDS Plot
meta_ord <- ordinate(physeq = percentages, method = "NMDS", distance = "bray")

plot_ordination(physeq = percentages, ordination = meta_ord)

View(metadata)
```

```
rownames(metadata) <- metadata$Sample # Using sample names as row names
percentages@sam_data <- sample_data(metadata) # Adding metadata to sam_data table
of phyloseq object percentages

meta_ord <- ordinate(physeq = percentages, method = "NMDS", distance = "bray") #
Calculating beta diversity
plot_ordination(physeq = percentages, ordination = meta_ord, color = "Location") #
Plotting beta diversity

#PCoA Plot
pcoa <- ordinate(physeq = percentages, method = "PCoA", distance = "bray")
plot_ordination(physeq = percentages, ordination = pcoa, color = "Location")

#EXPLORING DEEPER
percentages_glom <- tax_glom(percentages, taxrank = 'Phylum')
View(percentages_glom@tax_table@.Data)
percentages_df <- psmelt(percentages_glom)
str(percentages_df)
View(percentages_df)
write.csv(percentages_df, file = "$PATH")

absolute_glom <- tax_glom(physeq = all_biomdata_bacteria_1, taxrank = "Phylum")
absolute_df <- psmelt(absolute_glom)
str(absolute_df)

absolute_df$Phylum <- as.factor(absolute_df$Phylum)
install.packages("RColorBrewer")

library(RColorBrewer)

phylum_colors_abs <-
colorRampPalette(brewer.pal(8,"Dark2"))(length(levels(absolute_df$Phylum)))

absolute_plot <- ggplot(data= absolute_df, aes(x=Sample, y=Abundance,
fill=Phylum))+
  geom_bar(aes(), stat="identity", position="stack")+
  scale_fill_manual(values = phylum_colors_abs)

percentages_df$Phylum <- as.factor(percentages_df$Phylum)
phylum_colors_rel <- colorRampPalette(brewer.pal(8,"Dark2"))
(length(levels(percentages_df$Phylum)))
relative_plot <- ggplot(data=percentages_df, aes(x=Sample, y=Abundance,
fill=Phylum))+
  geom_bar(aes(), stat="identity", position="stack")+
  scale_fill_manual(values = phylum_colors_rel)

absolute_plot #Loading the plot
relative_plot #Loading the plot

#Giving cutoff value for visualization
```

```
percentages_df$Phylum <- as.character(percentages_df$Phylum)
percentages_df$Phylum[percentages_df$Abundance < 5] <- "Phyla < 5% abund."
unique(percentages_df$Phylum)

percentages_df$Phylum <- as.factor(percentages_df$Phylum)
phylum_colors_rel<- colorRampPalette(brewer.pal(8,"Dark2"))
(length(levels(percentages_df$Phylum)))
relative_plot <- ggplot(data=percentages_df, aes(x=Sample, y=Abundance,
fill=Phylum))+
  geom_bar(aes(), stat="identity", position="stack")+
  scale_fill_manual(values = phylum_colors_rel)

#Each taxa level plot
#Plot [Taxa_Name]
Desul <- subset_taxa(all_biomdata_bacteria_1, Phylum == "[Taxa_Name]")
unique(Desul@tax_table@.Data[,2])

Desul_percentage <- transform_sample_counts(Desul,function(x) x*100 / sum(x))

Desul_df$Genus[Desul_df$Abundance < 2] <- "Genera < 2% Abund."
Desul_df$Genus <- as.factor(Desul_df$Genus)
genus_color_Desul <-
colorRampPalette(brewer.pal(8,"Dark2"))(length(levels(Desul_df$Genus)))

Plot_Desul <- ggplot(data = Desul_df, aes(x=Sample, y=Abundance, fill=Genus))+
  geom_bar(aes(), stat = "identity", position = "stack")+
  scale_fill_manual(values = genus_color_Desul)
Plot_Desul

#JUST FOR CHECKING
View(Desul_df)
View(Desul_percentage@tax_table@.Data)
View(Desul_glom@tax_table@.Data)
View(all_biomdata_bacteria_1@tax_table@.Data)
```

```
#Script for Archaea analysis in R
# Load required packages, input data from kraken-biom result
if (!requireNamespace("BiocManager", quietly = TRUE))
  install.packages("BiocManager")
BiocManager::install("phyloseq") # Install phyloseq
install.packages(c("ggplot2", "readr", "patchwork")) #install ggplot2 and patchwork
to chart publication-quality plots and readr to read rectangular datasets.
library("phyloseq") # load library
library("phyloseq") # load library
library("ggplot2") #load library
library("readr") #load library
library("patchwork") #load library

# Load data with the number of reads per OTU and taxonomic labels for each OTU
setwd("$PATH") # tell R in which directory we are working
all_biomdata <- import_biom("all.biom")
class(all_biomdata) # The "class" command indicate that we already have our
phyloseq object
View(all_biomdata@tax_table@.Data)
View(all_biomdata@otu_table@.Data)

#Load another package
library(phyloseq)
library(biomformat)

# Add sample name
new_sample_names <- c("115 m", "1457 m", "100 m")
sample_names(all_biomdata) <- new_sample_names

# Adjustment taxa name
all_biomdata@tax_table <- substring(all_biomdata@tax_table, 4) # detele the 4
letter of tax_table
colnames(all_biomdata@tax_table)<- c("Kingdom", "Phylum", "Class", "Order",
                                     "Family", "Genus", "Species") # rename the
"Rank" with the taxonomic order

all_biomdata_archaea <- subset_taxa(all_biomdata, Kingdom == "Archaea") # Filter
all data samples for Archaea.
View(all_biomdata_archaea@tax_table@.Data)
View(all_biomdata_archaea@otu_table@.Data)

# Using glom
glom <- tax_glom(all_biomdata_archaea, taxrank='Genus')
glom_phylum <- tax_glom(all_biomdata_archaea, taxrank = "Phylum")

# Convert to csv
dat <- psmelt(glom)
dat2 <- psmelt(all_biomdata_bacteria)
dat_phylum <- psmelt(glom_phylum)
```

```
write.csv(dat, file='$PATH')
write.csv(dat_phylum, file = "$PATH")

# Counting phyla
unique(all_biomdata_archaea@tax_table@.Data[,"Phylum"])

#EXPLORING ABUNDANCE TABLE
View(all_biomdata_archaea@otu_table@.Data)
write.csv(all_biomdata_archaea@otu_table@.Data, file = "$PATH")

#ALPHA DIVERSITY PLOT
all_biomdata_archaea #Checking OTU and Taxonomy Table
sample_sums(all_biomdata_archaea) #Count total sample for each group
summary(all_biomdata@otu_table@.Data)

richness_diversity <- estimate_richness(all_biomdata_archaea, measures =
c("Observed", "Shannon","Simpson"))
View(richness_diversity)

richness_diversity_df <- as.data.frame(richness_diversity)
richness_diversity_df

write.csv(richness_diversity, file = "$PATH")

plot_richness(physeq = all_biomdata_archaea,
              measures = c("Shannon","Simpson"))

#Absolute and Relative Abundances
summary(all_biomdata_archaea@tax_table@.Data== "")

all_biomdata_archaea_1 <- subset_taxa(all_biomdata_archaea, Genus != "")
summary(all_biomdata_archaea_1@tax_table@.Data== "")
head(all_biomdata_archaea_1@otu_table@.Data)

percentages <- transform_sample_counts(all_biomdata_archaea_1, function(x) x*100 /
sum(x) )
head(percentages@otu_table@.Data)

#BETA DIVERSITY
distanceMethodList
metadata <- data.frame(Sample=c("115 m", "1457 m", "100 m"), Location=c("115 m",
"1457 m", "100 m")) # Making dataframe with metadata

#NMDS Plot
meta_ord <- ordinate(physeq = percentages, method = "NMDS", distance = "bray")

plot_ordination(physeq = percentages, ordination = meta_ord)

View(metadata)
rownames(metadata) <- metadata$Sample # Using sample names as row names
```

```
percentages@sam_data <- sample_data(metadata) # Adding metadata to sam_data table
of phyloseq object percentages

meta_ord <- ordinate(physeq = percentages, method = "NMDS", distance = "bray") #
Calculating beta diversity
plot_ordination(physeq = percentages, ordination = meta_ord, color = "Location") #
Plotting beta diversity

#PCoA Plot
pcoa <- ordinate(all_biomdata_archaea_1, method = "PCoA", distance = "bray")
plot_ordination(physeq = percentages, ordination = pcoa, color = "Location")


#EXPLORING DEEPER
percentages_glom <- tax_glom(percentages, taxrank = 'Phylum')
View(percentages_glom@tax_table@.Data)
percentages_df <- psmelt(percentages_glom)
str(percentages_df)

absolute_glom <- tax_glom(physeq = all_biomdata_archaea_1, taxrank = "Phylum")
absolute_df <- psmelt(absolute_glom)
str(absolute_df)

absolute_df$Phylum <- as.factor(absolute_df$Phylum)
install.packages("RColorBrewer")

library(RColorBrewer)

phylum_colors_abs <-
colorRampPalette(brewer.pal(8,"Dark2"))(length(levels(absolute_df$Phylum)))

absolute_plot <- ggplot(data= absolute_df, aes(x=Sample, y=Abundance,
fill=Phylum))+
  geom_bar(aes(), stat="identity", position="stack")+
  scale_fill_manual(values = phylum_colors_abs)

percentages_df$Phylum <- as.factor(percentages_df$Phylum)
phylum_colors_rel <- colorRampPalette(brewer.pal(8,"Dark2"))
(length(levels(percentages_df$Phylum)))
relative_plot <- ggplot(data=percentages_df, aes(x=Sample, y=Abundance,
fill=Phylum))+
  geom_bar(aes(), stat="identity", position="stack")+
  scale_fill_manual(values = phylum_colors_rel)

absolute_plot #Loading the plot
relative_plot #Loading the plot

#Giving cutoff value for visualization
percentages_df$Phylum <- as.character(percentages_df$Phylum)
percentages_df$Phylum[percentages_df$Abundance < 5] <- "Phyla < 5% abund."
```

```
unique(percentages_df$Phylum)

percentages_df$Phylum <- as.factor(percentages_df$Phylum)
phylum_colors_rel<- colorRampPalette(brewer.pal(8,"Dark2"))
(length(levels(percentages_df$Phylum)))
relative_plot <- ggplot(data=percentages_df, aes(x=Sample, y=Abundance,
fill=Phylum))+
  geom_bar(aes(), stat="identity", position="stack")+
  scale_fill_manual(values = phylum_colors_rel)

relative_plot

View(all_biomdata_archaea_1@tax_table@.Data)

#Each taxa level plot
#Plot [Taxa_Name]
Desul <- subset_taxa(all_biomdata_archaea_1, Phylum == "[Taxa_Name]")
unique(Desul@tax_table@.Data[,2])

Desul_percentage <- transform_sample_counts(Desul,function(x) x*100 / sum(x))
Desul_glom <- tax_glom(Desul_percentage, taxrank = "Genus")
Desul_df <- psmelt(Desul_glom)
Desul_df$Genus[Desul_df$Abundance < 10] <- "Genera < 10.0 Abund."
Desul_df$Genus <- as.factor(Desul_df$Genus)
genus_color_Desul <-
colorRampPalette(brewer.pal(8,"Dark2"))(length(levels(Desul_df$Genus)))

Plot_Desul <- ggplot(data = Desul_df, aes(x=Sample, y=Abundance, fill=Genus))+
  geom_bar(aes(), stat = "identity", position = "stack")+
  scale_fill_manual(values = genus_color_Desul)
Plot_Desul

View(Desul_df)
```
